# Morphology and Properties of Polyolefin Elastomer/Polyamide 6/Poly(lactic Acid) In Situ Special-Shaped Microfibrillar Composites: Influence of Viscosity Ratio

**DOI:** 10.3390/polym14214556

**Published:** 2022-10-27

**Authors:** Min Shi, Lijun Wang, Jing Sun, Wensheng Yang, Hui Zhang

**Affiliations:** 1 State Key Laboratory of Chemical Resource Engineering, Beijing University of Chemical Technology, Beijing 100029, China; 2 Guizhou Material Industrial Technology Institute, Guiyang 550014, China

**Keywords:** in situ microfibrillar composites, polyolefin elastomer, poly(lactic acid)/polyamide 6, special-shaped microfibrils, viscosity ratio

## Abstract

In Situ microfibrillation is an easy and economical processing method, which has drawn wide concern in recent years. In Situ special-shaped microfibrillar composites, which with poly(lactic acid)/polyamide 6 (PA6/PLA) together formed special-shaped microfibrils in polyolefin elastomer (POE) matrix, were successfully prepared by using multistage stretching extrusion technology. Four types of PA6 with different viscosity were utilized to investigate the effect of viscosity ratio of PA6 to PLA on the structure evolution of special-shaped microfibrils and the mechanical properties of POE/(PA6/PLA) composites. The morphological observation showed that the viscosity ratio was closely associated to the size and shape of PA6 and greatly affected the microfibrillar morphology of PLA/PA6. When the viscosity ratio of PA6 to PLA was less than 2.2, the “gourd-skewers-like” structure microfibrils were obtained. When the viscosity ratio of PA6/PLA to 14.2 was further increased, the “trepang” structure microfibrils were dominant. The “gourd skewers” structure microfibrils were favorable to improvement the tensile strength, Young’s modulus, and viscoelastic properties of POE/(PA6/PLA) blends compared to the “trepang” structure microfibrils. In addition, the morphology of microfibrils exhibited a negligible effect on the melting and crystallization temperature and crystallization degree of PLA and POE matrix. This work provides a new strategy for designing the in situ special-shaped microfibrillar composites with improved mechanical properties.

## 1. Introduction

The conventional composite reinforced by the adding of a reinforcing filler (e.g., glass fiber or carbon filler) is typically an organic-inorganic system [1,2]. The adding of filler can effectively improve the mechanical properties of matrix material, which are increasingly attracting the interest of researchers [3,4,5]. In Situ microfibrillar composites (MFCs) are a type of polymer-polymer composites, in which the dispersed component develop microfibrils in situ and distribute uniformly in the matrix during the blending process [6,7,8,9]. MFCs, providing a potential, economical way of tailoring the properties of the polymer blend’s product according to the desired application, which has been widely concerned in recent years [10,11,12]. Xie et al. [13] prepared the Poly(lactic acid) (PLA)/Poly (butylene succinate) (PBS) MFCs and found that the dispersed phase PBS developed in situ nanofibrillar PBS and dramatically improved the strength, modulus, and ductility of composites compared with pure PLA. This phenomenon was found in many composites systems, such as PLA/polyamide 6 (PA6) composites [14], Polyolefin elastomer (POE)/poly(trimethylene terphthalate) (PTT) composites [15,16], and poly(ethylene terephthalate) (PET)/isotatic polypropylene(iPP) composites [17]. Sun et al. [18] prepared POE/PLA MFCs and found that the POE/PLA composites appeared double yield behavior during the tensile process, which was beneficial to improve the mechanical properties of MFCs.

When the mixed material were fed into the extruder, a series of complex changes will occur [19,20]. Polymers will undergo different morphological changes. The dispersed component may breakup into small droplets under the action of the shear and stretching stresses [21,22]. The interfacial tension will drive dispersed droplets form a spherical shape, while the viscous force will drive the drawing of the droplets. When the viscous force was larger than the interfacila tension, the dispersed droplet deformed, making the dispersed droplets transform from droplets to the final fibril [23,24,25,26]. The formation of in situ fibrils is usually an overall result of continuous deformation, coalescence, elongation, and orientation of dispersed droplets [27,28]. The final morphology of the dispersed phase in the blends greatly affects the physical and mechanical properties of MFCs [29,30,31,32]. There are many factors affecting the final morphology of dispersed phase in the MFCs, which include viscosity ratio [33,34], composition [35,36,37], stretch ratio [38,39,40], and compatibility of components [41,42]. It has been reported that the viscosity ratio (*η_d_/η_m_*, where *η_d_* is the viscosity of the dispersed component and *η_m_* is the viscosity of the matrix) of the blend played an important role on the formation of the fibril shape [43,44]. Low viscosity ratio facilitated the formation of smaller and more uniform dispersed phase particles, and then led to a narrow diameter distribution of microfibrils after stretching, which resulted in higher strength and modulus [45,46,47,48]. Zhao et al. [49] employed PP with different melt flow rates (MFRs, 3.6, 5, 19, 36 g per 10 min, 230 °C per 2.16 kg) as the matrix prepared PP/PET MFCs and investigated the effect of PP’s viscosity and the PET concentration on the PET nanofibril formation. They found that a PP with a lower melt flow rate (MFRs) was better for generating a higher aspect ratio and smaller diameter PET nanofibrils. The diameter of fibrils from a higher MFR PP decreased with an increasing PET concentration. Yi et al. [48] prepared the isotactic polypropylene(iPP) and poly (ethylene terephthalate) (PET) MFCs and investigated the effect of the viscosity ratio on the morphology and mechanical properties of iPP/PET microfibrillar blend. They found that the viscosity ratio played an important role in the size of dispersed phase, and subsequently affected the morphology of PET microfibrils during stretching. The appropriate viscosity ratio for improved mechanical properties was about 0.083. Min et al. [50] investigated the relationship between the viscosity ratio and morphology of polyethylene (PE)/polystyrene (PS) blend extrudates, and found that the droplets became undulant fibers when the 0.7 < *η_d_*/*η_m_* < 1.7, while *η_d_*/*η_m_* was greater than 2.2, undeformed droplets are observed. However, the viscosity ratio in these studies was the dispersed component viscosity to the matrix component viscosity. In our previous work, in situ special-shaped microfibrillar composites with PLA and PA6 special-shaped microfibrils in POE matrix were first successfully prepared. How the viscosity ratio between the dispersed component (PLA and PA6) affected morphology evolution of MFCs needs to be investigated in more detail.

Herein, in this study, the PA6/PLA master batch with different PA6 viscosity was chosen as a dispersed phase to prepare the POE-based MFCs through the technology of multistage stretching extrusion. The influence of the viscosity ratio of PA6 to PLA on PA6/PLA special-shaped microfibrillar morphology evolution was examined. In addition, the mechanical, rheological, and crystallization properties affected by the morphology of special-shaped microfibrils were carefully investigated.

## 2. Experimental

### 2.1. Materials

POE (ENGAGE 8200) with a density of 0.87 g/cm^3^ and a melt index of 5.0 g/10 min (190 °C) was purchased from Dow Chemical Company, Knoxville, TN, USA. PLA (4032D) with a L-lactide content of roughly 98% and a density of 1.24 g/cm^3^ was purchased from Natureworks Company, Plymouth, MN, USA. PA6 with different relative viscosity was purchased from Xinhui meida Nylon Co., Ltd., Jiangmen, China. The physical parameters of POE and PLA were given in Table 1 and the physical parameters of PA6 was given in Table 2.

### 2.2. Specimen Preparation

First, PLA and PA6 were dried in a vacuum oven at 80 °C and 100 °C, respectively, for 12 h. Then, the PA6/PLA master batches with weight ratios of 1/1 were extruded by the twin-screw extruder (CTE-20, Nanjing Jieya Extruded Equipment Co., China). The screw speed was 200 rpm and the temperatures from the hopper to the exit of the extruder were 225–230 °C. Finally, the POE/(PLA/PA6) composites with the weight ratio of 75/25 were extruded by the multistage stretching extrusion assembly, which contained a twin-screw extruder (SHJ-30, Nanjing Jieya Extruded Equipment Co., China), an extrusion die with an assembly of laminating–multiplying elements (LMEs). The screw speed was 250 rpm and the temperature from the hopper to the exit of the extruder was 50–230 °C. The temperature of LMEs was 230 °C. After the POE/(PLA/PA6) melt flowed from the extruder die, the extrudate was further stretched at rate of 80 rpm to obtain MFCs. The in situ POE/(PA6/PLA) MFCs with different viscosity ratio (the ratio of PA6 viscosity to the PLA viscosity at the same temperature) were coded as MFCs-xx, where xx represented the viscosity ratio of PA6 to PLA.

### 2.3. Characterization

A field emission scanning electron microscope (SEM, QUANTA FEG 250, FEI Company, USA) was employed to observe the microstructure of the composites. The composite sheets were placed in liquid nitrogen for 2 h, finally the samples were cryogenically fractured along to extrusion direction or perpendicular to extrusion direction. The smooth fractured surfaces can then be taken for SEM observation or the further etching process. For the etching, the fractured surfaces were immersed in hot xylene at 80 °C for 12 h to dissolute POE, after which it was cleaned by ethyl alcohol, and then immersed in dichloromethane (CH_2_Cl_2_) at 20 °C for 1 h to dissolute PLA. Note that all etched surfaces were cleaned by using distilled water prior to SEM observation.

Thermal analysis of the samples were studied by DSC (Q10, TA Company, New Castle, DE, USA) under N_2_ atmosphere. The dynamic scanning was applied at a heating rate of 10 °C/min from 5 °C to 240 °C for MFCs. After holding the sample at a high temperature for 5 min, it was then cooled down to 5 °C at a heating rate of 10 °C/min to analyze the crystallinity behavior.

The crystallinity of PA6 and POE were calculated using the following equation:

(1) $$X_{c}=\frac{\Delta H_{m}}{\varphi \times \Delta H_{m}^{0}}\times 100\%$$

The crystallinity of PLA was calculated using the following equation:

(2) $$X_{c}=\frac{\Delta H_{m}-\Delta H_{c}}{\varphi \times \Delta H_{m}^{0}}\times 100\%$$

In Equations (1) and (2), $\Delta H_{m}$ and $\Delta H_{c}$ were the enthalpies of melt crystallization and cold crystallization, respectively. The $\Delta H_{m}^{0}$ was the melting enthalpy for 100% crystallinity. The $\Delta H_{m}^{0}$ values of PA6, PLA, and POE were 230 J/g [51], 93.7 J/g [52], and 205 J/g [53], respectively. The symbol φ represented the mass percentage of the PA6, PLA, or POE in the MFCs.

The dynamic rheological experiments of PLA, PA6, and POE/(PLA/PA6) MFCs were studied by Rotational rheometer (MARS60, USA), using parallel-plate geometry with a diameter of 25 mm. The dynamic viscoelastic properties were determined with frequencies from 0.05 to 100 rad/s at 1% strain amplitude. The measured temperature of PLA and PA6 were fixed at 230 °C, which was the processing temperature. The measured temperature of POE/(PLA/PA6) MFCs was fixed at 130 °C, which was lower than the melting temperature of PLA/PA6. According to ASTM standard D638, tensile tests were made by a universal testing machine (CMT6104, China) with a crosshead speed of 500 mm/min and a gauge length of 25 mm. Specimens for tensile test were cut from the extrusion sheet along or perpendicular to the extrusion direction. The width and the thickness of specimens were measured before tensile test. All measurements were repeated at least five times and the average values were reported.

## 3. Results and Discussion

### 3.1. Viscoelastic Behavior of PA6 and PLA

**Figure 1:**
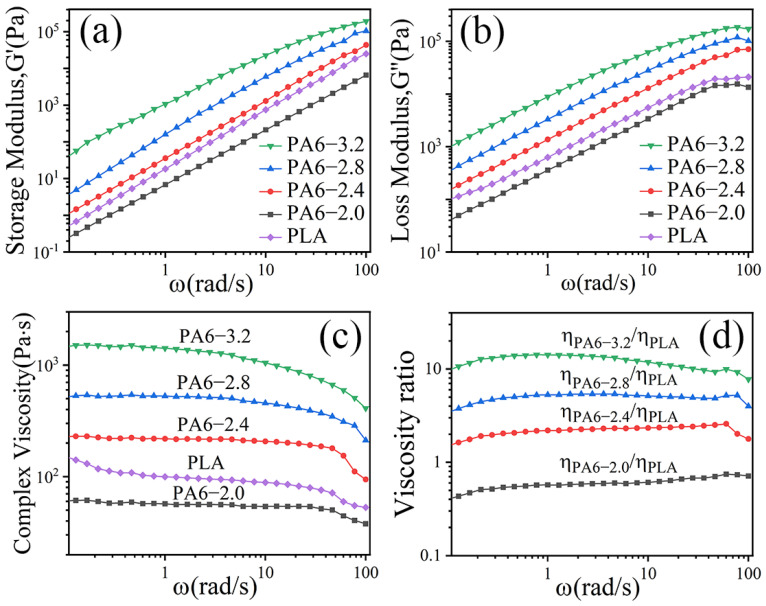
Viscoelastic behavior of PA6 and PLA at 230 °C and viscosity ratio of PA6/PLA: (**a**) storage modulus (G′), (**b**) loss modulus (G″), (**c**) complex viscosity (η*), and (**d**) viscosity ratio of PA6/PLA.

Figure 1 shows the storage modulus(G′), loss modulus(G″), and complex viscosity(η*) of PA6 and PLA and the viscosity ratio of PA6/PLA as a function of frequency. It can be seen that the G′ of PA6 increased with the increasing of viscosity of PA6 at identical frequencies. These results can be attributed to the restriction of the PA6 chain’s mobility, which can improve the capability of storing the deformation energy over extended periods of time (increasing the relaxation time) [14]. The G″ of PA6 increased with the increasing of PA6 viscosity (Figure 1b). This can be attributed to the increasing of energy consumption of PA6 chain’s slippage in unit time [40]. In addition, with the increasing of frequencies, the η* of PA6 and PLA decreased continuously (Figure 1c), implying that the PA6 and PLA were non-Newtonian fluids and all followed the shear thinning behavior [34]. When the frequency was 1, the viscosity ratio of PA6 to PLA was 0.5, 2.2, 5.3, and 14.2. The G′, G″, and η* of PLA was only greater than the PA6-2.0 at identical frequencies and the viscosity ratio of PA6-2.0/PLA was lower than 1.

### 3.2. Morphology and Microstructure of MFCs

**Figure 2:**
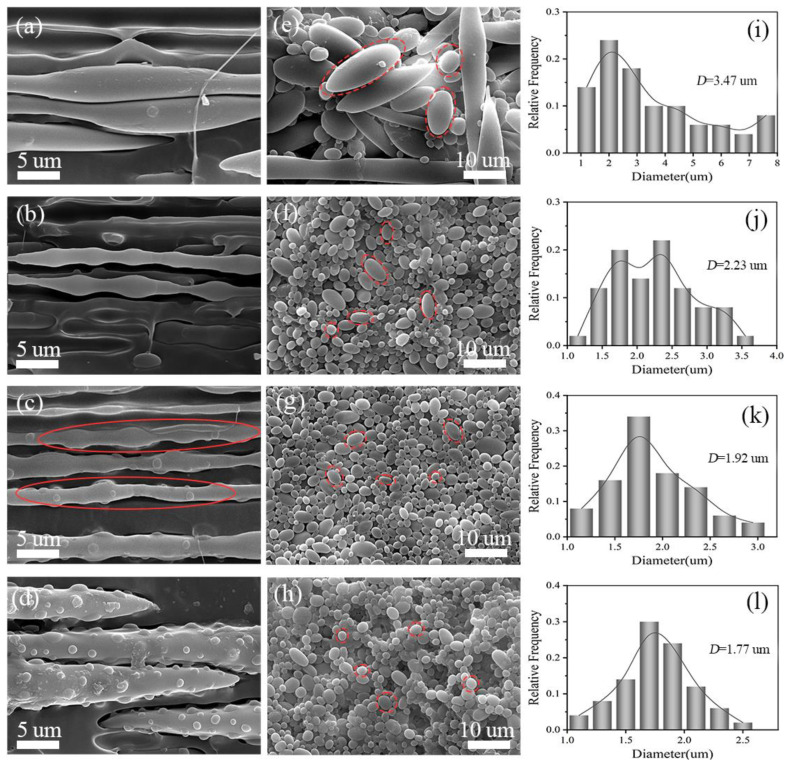
(**a**–**d**) SEM images of in situ POE/(PLA/PA6) composites and (**e**,**f**) the PLA/PA6 microfibrils after etching the PLA, (**i**–**l**) Quantitative analyses of the PA6 phase after etching the PLA. (**a**,**e**,**i**) MFCs-0.5, (**b**,**f**,**j**) MFCs-2.2, (**c**,**g**,**k**) MFCs-5.3, (**d**,**h**,**l**) MFCs-14.2.

Figure 2 shows the SEM images of POE/(PA6/PLA) in situ MCFs and the PA6/PLA microfibrils after etching the PLA and the quantitative analyses of the PA6 phase after etching the PLA. From Figure 2a–d, it can be seen that, after hot stretching, the dispersed phase (PA6 and PLA) was fully extended into special-shaped structure microfibrils in the POE matrix. As compared with the regular cylinder-shaped microfibrils in the POE/PLA composites [18], the special-shaped microfibrils had an uneven diameter. When the viscosity ratio of PA6/PLA was 0.5, the gourd-skewers-like structure microfibrils were dominance. The gourd-skewers-like microfibrils had many knots with a larger diameter connected by the skewer with smaller diameter. With the viscosity ratio of PA6 to PLA increased, the diameters of knots and the distance between knots all tended to decrease. When the viscosity ratio of PA6 to PLA increased to 5.3, the microfibrillar morphology appeared in “trepang” structure (red dotted circle). Further increasing the viscosity ratio of PA6/PLA to 14.2, the “trepang” structure microfibrils were dominant. The trepang-like microfibrils had a large diameter with many small knots on their surfaces. From Figure 2e–h, it can be seen that when the viscosity ratio of PA6 to PLA was 0.5, the PA6 phase presented short rod-like microfibrils, ellipsoid particle, and spherical particle after etching PLA. With the increasing of PA6 viscosity, spherical particles were dominant and the diameter of ellipsoid particle and spherical particles decreased gradually (red dotted circle). The average diameter of PA6 decreased from 3.47 μm to 1.77 μm and the diameter distribution of PA6 became narrow with the increasing of viscosity of PA6 (Figure 2i–l), revealing that the deformation degree of PA6 phase became lower with the increasing of viscosity of PA6 during the hot stretching.

**Figure 3:**
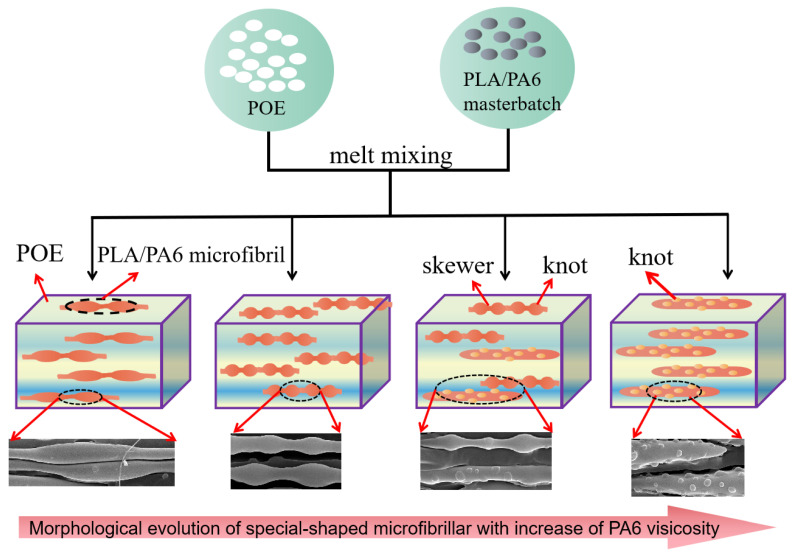
Schematic diagram for the evolution of special-shaped microfibrils.

The evolution mechanism of special-shaped microfibrils was shown in Figure 3. when the PA6/PLA master batches were feed into the twin screw extruder, the particles of PA6/PLA master batches broke up to form droplets under the action of shear stress. Under the action of the shear and stretch stress, the dispersed droplets occurred deformed, making the dispersed transformed from droplets to the final fibril [19,27]. When the viscosity ratio of PA6 to PLA was 0.5, due to the PA6 generated short rod-like microfibrils and ellipsoid particle with a larger diameter during the hot stretching, the gourd-skewers-like microfibrils had knots with larger diameter and length. With the viscosity ratio of PA6 to PLA increasing to 2.2, the diameter and length of knots of the gourd-skewers-like microfibrils decreased due to diameter of the PA6 particles decreased. Further increasing the viscosity ratio of PA6/PLA to 14.2. the microfibrils morphology turned into the “trepang” structure.

### 3.3. Rheological Characterization

**Figure 4:**
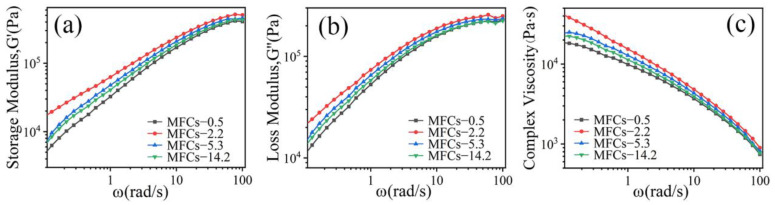
Viscoelastic behavior of POE/(PA6/PLA) composites with different viscosity ratio of PA6 to PLA. (**a**) Storage Modulus(G′), (**b**) Loss Modulus(G″), (**c**) Complex Viscosity(η*).

Figure 4 shows the dependence of storage modulus(G′), loss modulus(G″), and complex viscosity (η*) on angular frequency(ω) for in situ POE/(PA6/PLA) MFCs at 130 °C. It was evident that the G′ and G″ of all the specimens increased with increasing angular frequency (Figure 4a,b). At low frequencies, the G′, G″ of POE/(PA6/PLA) blends increased linearly. At high frequencies, the frequency-dependence of G′, G″ was substantially weakened and a small platform appeared due to the frequency’s response difference of PA6/PLA microfibrils physically entangled network. The information of situ microfibrils made effective contributions for not only the elastic behavior, but also the viscous behavior. Sun et al. [18] had the similar conclusion about the research of POE/PLA microfibrillar composites fabricated through multistage stretching extrusion. From Figure 4c, it can be seen that all the specimens exhibited shear thinning behavior, indicating that the in situ POE/(PA6/PLA) blends were non-Newtonian fluids, which was similar to the POE/PTT microfibrillar composites [15]. With the increasing of ω, the η* of POE/(PA6/PLA) blends decreased continuously. This result can be ascribed to the de-entanglement of POE chain and the PA6/PLA microfibril remain highly oriented (PA6/PLA microfibrils were solid-state at 130 °C). In addition, with the increasing of viscosity ratio of PA6 to PLA, the G′, G″ and η* of POE/(PA6/PLA) blends increased firstly and then decreased at the same ω. When the viscosity ratio of PA6 to PLA was 2.2, the viscoelastic properties of POE/(PA6/PLA) blends was maximum, revealing that the “gourd skewers” structure microfibrils with a small diameter and length of knots was favorable to improvement the viscoelastic properties of POE/(PA6/PLA) blends than the “trepang” structure microfibrils. The viscoelastic properties of PA6-3.2 were maximum (Figure 1). On the contrary, the minimum value of viscoelastic properties were obtained at the viscosity ratio of 0.5, which was due to the decreased of PA6-2.0 viscoelastic properties (Figure 1) and the larger diameter of gourd-skewers structure microfibrils.

### 3.4. Thermal Characterization

**Figure 5:**
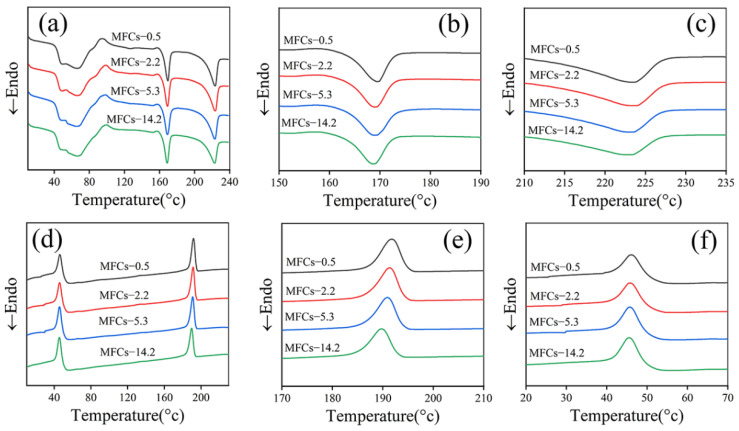
(**a**–**c**) DSC heating curves and (**d**–**f**) DSC cooling curves of situ POE/(PA6/PLA) MFCs, (**b**,**c**) magnification of heating curves, (**e**,**f**) magnification of cooling curves.

**Figure 6:**
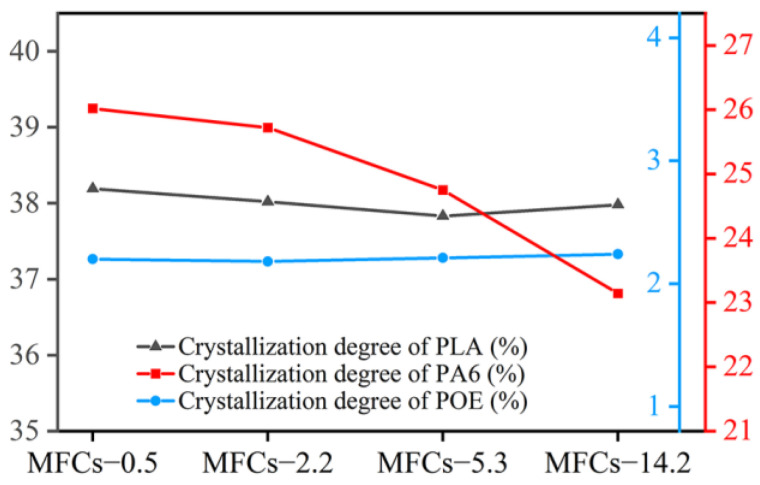
Crystallization degree of PLA, PA6 and POE.

Figure 5 shows the DSC curves of in situ POE/(PA6/PLA) MFCs. It can be seen that the peak melting temperature of PLA at about 169 °C and that of PA6 at about 223 °C were not affected by the viscosity ratio of PA6/PLA. In addition, the melting enthalpy of PLA was almost constant, while that of PA6 decreased with increasing the viscosity ratio of PA6/PLA. These results indicated that the morphology of PLA/PA6 microfibrils (“gourd skewers” or “trepang”) had negligible effect on the melting temperature of both PLA and PA6. Due to the poor crystallization kinetics of pure PLA, there was not crystallization behavior during the cooling process (Figure 5d) [54]. However, an obvious cold crystallization peak with the peak temperature of about 100 °C can be seen during the heating cycle, which was used to calculate the crystallization degree of PLA. In addition, the crystallization temperature of PA6 decreased, while that of POE were almost constant with the viscosity ratio of PA6/PLA increased (Figure 5e–f). This result indicated that the morphology of PLA/PA6 microfibrils had negligible effect on the melting temperature of matrix POE. The decreased of crystallization temperature of PA6 can be ascribed to the decreased of the PA6 molecular chain’s the mobility with the increasing viscosity of PA6. Figure 6. shows the crystallization degree of PLA, PA6, and POE in MFCs. It can be seen that the crystallization degree of PLA was 28.0% and POE was 2.2%, respectively, and were hardly affected by the viscosity ratio of PA6/PLA. In addition, the crystallization degree of PA6 decreased with the viscosity of PA6 increasing. These results can be ascribed to the increased of the restrictions effect of crystal nucleus formation with the increasing viscosity of PA6.

### 3.5. Mechanical Property

**Figure 7:**
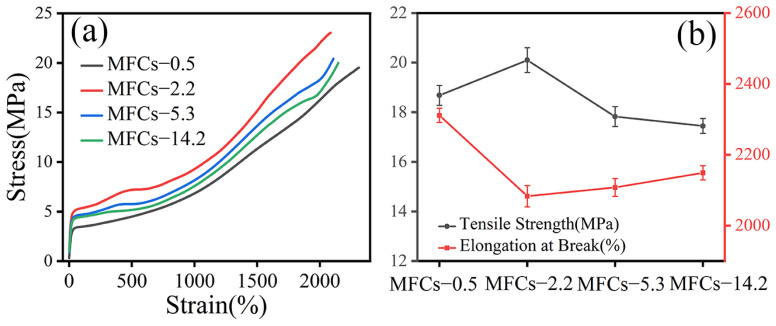
Typical stress-strain curves and tensile properties of in situ POE/(PA6/PLA) composites along to extrusion direction. (**a**) Stress-strain curves, (**b**) Tensile properties.

Figure 7 shows the stress-strain curves and tensile properties of POE/(PA6/PLA) MFCs along to extrusion direction. It was seen that the stress-strain curve of POE/(PA6/PLA) blends showed typical response of elastomeric materials. With the increasing of viscosity ratio of PA6/PLA, the special-shaped microfibrillar composites appeared multiple-yield behavior during the tensile test. As shown in Figure 7b, the tensile strength of MFCs increased firstly and then decreased with the viscosity ratio of PA6/PLA, which agreed with Young’s modulus of POE/(PA6/PLA) (Figure 7a). When the viscosity ratio of PA6/PLA was 2.2, the POE/(PA6/PLA) MFCs had the highest tensile strength and Young’s modulus. This result revealed that “gourd skewers” with small diameter and length of knots were favorable to improvement the strength and modulus of POE/(PA6/PLA) blends compared to the “trepang” structure microfibrils. In addition, the elongation at break of MFCs decreased firstly and then increased with increasing the viscosity ratio of PA6/PLA. When the viscosity ratio of PA6/PLA was 0.5, the maximum value of elongation at break can be obtained. This result can be ascribed to the large specific surface area of “gourd skewers” was favorable to the transfer of stress.

**Figure 8:**
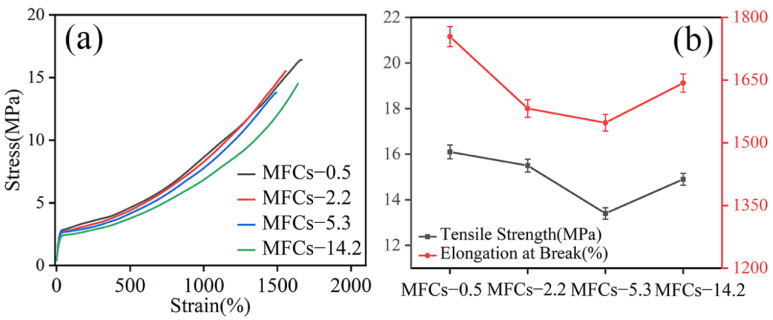
Typical stress-strain curves and tensile properties of in situ POE/(PA6/PLA) composites perpendicular to extrusion direction. (**a**) Stress-strain, (**b**) Tensile properties.

Figure 8 shows the stress-strain curves and tensile properties of POE/(PA6/PLA) MFCs perpendicular to extrusion direction. It was seen that the stress-strain curve of POE/(PA6/PLA) blends showed typical response of elastomeric materials. The POE/(PA6/PLA) MFCs had the similar stress-strain curves with increasing the viscosity ratio of PA6/PLA. As shown in Figure 8b, the tensile strength and elongation at break of MFCs decreased firstly and then increased with the increase of viscosity ratio of PA6/PLA. When the viscosity ratio of PA6/PLA was 0.5, the tensile strength and elongation at break of POE/(PA6/PLA) MFCs obtained a maximum value. This result can be ascribed to enhance of interfacial adhesion between microfibrils and POE matrix due to the large specific surface area of “gourd skewers”.

## 4. Conclusions

In this work, the POE/(PA6/PLA) MFCs based on the bends of POE as matrix and PA6/PLA master batches as dispersed phase were successfully fabricated by using multistage stretching extrusion technology. The viscosity ratio of PA6 to PLA plays an important role in the morphology of PA6/PLA microfibrils during stretching. When the viscosity ratio of PA6/PLA was less than 2.2, the “gourd-skewers-like” structure microfibrils were obtained. Further increased the viscosity ratio of PA6/PLA to 14.2, the “trepang” structure microfibrils were obtained. The “gourd skewers” microfibrils with small diameter were favorable to improvement mechanical properties and viscoelastic properties of POE/(PA6/PLA) blends compared to the “trepang” structure microfibrils.

## Figures and Tables

**Table 1:** Physical parameters of POE and PLA.

Sample	Density (g/cm^3^)	Melting Index (g/10 min)	Tensile Strength at Break (MPa)	Tensile Elongation at Break (%)
POE 8200	0.87	5.0 (190 °C)	9.3	>1000
PLA 4032D	1.24	7.0 (210 °C)	53	6

**Table 2:** Physical parameters of PA6.

Sample	PA6-2.0	PA6-2.4	PA6-2.8	PA6-3.2
Material	M2000	M2400	M2800	M3400
Relative Viscosity	2.07	2.46	2.81	3.28
Density (g/cm^3^)	1.14	1.14	1.14	1.14
Melting Index (g/10 min)	137	34.6	15.2	6.5
Tensile Strength at Break (MPa)	66	61	65	64
Tensile Elongation at Break (%)	26	39	36	58

## Data Availability

The data that supports the findings of this study are available within the article.
